# Prophylactic Common Peroneal Nerve Release in Complex Total Knee Replacement: A Narrative Review of Rationale, Surgical Techniques, Outcomes, and Future Directions

**DOI:** 10.7759/cureus.90216

**Published:** 2025-08-16

**Authors:** Zubair Younis, Muhammad A Hamid, Faliq Abdullah

**Affiliations:** 1 Orthopaedics, The Royal Wolverhampton NHS Trust, Wolverhampton, GBR; 2 Orthopaedic Surgery, University Hospitals Birmingham NHS Foundation Trust, Birmingham, GBR; 3 Trauma and Orthopaedics, University Hospital of Wales, Cardiff, GBR

**Keywords:** nerve decompression, peroneal nerve palsy, prophylactic, total knee arthroplasty, valgus knee

## Abstract

Common peroneal nerve (CPN) palsy is a rare but serious complication following total knee arthroplasty (TKA), particularly in patients with severe valgus deformities and multiple comorbidities. Traditional soft tissue balancing techniques and postoperative precautions do not reliably prevent nerve injury in high-risk cases. Prophylactic common peroneal nerve release (PPNR) has emerged as a promising strategy to mitigate this risk by decompressing the nerve prior to deformity correction. This narrative review explores the anatomical and biomechanical basis for CPN vulnerability, the clinical rationale for PPNR, and its surgical technique. It also summarizes early clinical outcomes demonstrating preserved nerve function, faster rehabilitation, and minimal complications across multiple small series. Despite encouraging results, the current evidence is limited by small sample sizes. Future research should focus on establishing standardized indications, refining surgical protocols, and validating long-term outcomes through prospective trials. PPNR may represent a safe and effective adjunct in the management of complex valgus knees undergoing TKA, warranting broader consideration in high-risk patients.

## Introduction and background

Common peroneal nerve (CPN) palsy is a rare but debilitating complication of total knee arthroplasty (TKA), with reported incidence ranging from 0.3% to 10% in various series [1]. The risk is particularly heightened in patients with severe valgus deformity, combined flexion contractures, rheumatoid arthritis or pre-existing spinal pathology, or both at the time of TKA [2]. Additional comorbidities associated with increased nerve palsy risk secondary to TKA include obesity, increased age at surgery, and previous history of neuropathy [3]. Other risk factors include perioperative and intraoperative exposures, including anesthesia type, tourniquet time/pressure, traction, and compression. CPN injury may result in foot drop, sensory deficits, gait disturbance, and prolonged rehabilitation. These consequences are frequently distressing to patients and challenging to manage postoperatively [4].

While standard intraoperative precautions such as avoiding overcorrection, gentle manipulation, and postoperative bracing have been employed to minimize this risk, these conventional techniques do not reliably prevent nerve injury in all patients, particularly those with multiple risk factors [5].

Historically, peroneal nerve decompression has been used as a therapeutic measure after nerve injury has occurred, especially in cases of entrapment at the fibular neck [1]. In the field of limb lengthening and deformity correction, however, a proactive approach, prophylactic common peroneal nerve release (PPNR), has gained traction [6]. This technique, performed before acute deformity correction, aims to reduce nerve stretch and prevent postoperative palsy altogether. Its principles have now been extended to high-risk TKA cases. While PPNR is established in limb lengthening, the evidence base for its application specifically in TKA remains emerging and fragmented.

Biomechanical studies underscore the vulnerability of the CPN to stretch injury during deformity correction in TKA. Research indicates that nerve damage can occur at elongations as low as 4-11%, a threshold readily exceeded during the acute correction of severe valgus deformities, particularly when combined with flexion contracture [7]. This risk arises because the CPN, situated in the concavity of the deformity, undergoes substantial traction as the knee is realigned [5]. In TKA for valgus >15°, correction often requires lengthening the lateral structures by >2 cm, generating tensile forces that compromise microvascular perfusion and induce neuropraxia [5]. PPNR mitigates this by preemptively decompressing fibular tunnel constriction points and reducing baseline tension, thereby shielding the CPN from iatrogenic elongation beyond its physiological tolerance during correction [7].

PPNR has shown promising early results, with several studies reporting the complete preservation of nerve function and faster rehabilitation in patients undergoing TKA for valgus knees. This review synthesizes the current anatomical, biomechanical, and clinical evidence for PPNR in complex TKA, arguing for its consideration as a standard adjunct in patients with high-risk valgus deformity.

## Review

Anatomical and pathophysiological considerations

The CPN arises at the popliteal fossa apex from the sciatic nerve bifurcation. It courses inferolaterally along the biceps femoris, over the gastrocnemius' lateral head, issuing cutaneous branches to the leg [8]. At the fibular neck, it wraps superficially, separated from bone only by a thin epineurial layer, before passing through the peroneus longus attachments to divide into superficial and deep fibular nerves.

This anatomical vulnerability exposes the CPN to significant risk during valgus correction, where it endures elongation and compression [9]. The nerve, located in the concavity of the valgus and flexion deformity, undergoes acute lengthening during surgical correction, leading to neuropraxia. In these high-risk cases, the incidence of CPN palsy rises dramatically [5]. The nerve's limited mobility and superficial location make it particularly prone to neurapraxia in cases of rapid or aggressive correction. Xu et al. noted that pie-crusting techniques for lateral release can place the nerve as close as 13-35 mm from surgical instruments [7].

Incidence, risk factors, and consequences of CPN palsy

The overall incidence of CPN palsy after TKA ranges from 0.3% to 10%, with higher rates observed in patients with severe valgus deformities [3,4]. For valgus knees exceeding 10° deformity, the incidence rises to 0.4-4% and worsens dramatically with larger deformities; valgus ≥12° carries a 12-fold increased risk compared to milder cases [10,11]. PPNR prevented CPN palsy in all patients across three independent studies (pooled analysis of 59 knees), with no documented palsies during follow-up periods ranging from six months to 11 years [5,7,10].

Key risk factors include severe valgus deformity (thresholds: >15-20°), particularly when combined with flexion contracture, which exacerbates nerve stretching during correction [2,3]. Surgical factors like lateral soft tissue release (e.g., pie-crusting) pose direct injury risks [12]. Tourniquet use (prolonged time/pressure) and joint line elevation ≥5 mm further increase susceptibility [9,11,13]. Patient comorbidities, including pre-existing neuropathy (e.g., diabetic neuropathy), spinal pathology, rheumatoid arthritis, and prior knee surgery, also elevate risk [2,3]. 

CPN palsy causes motor deficits (foot drop, high-steppage gait) and sensory loss in the lateral leg/dorsal foot, severely impeding rehabilitation due to pain during knee motion [4]. Recovery is often incomplete, ~50% of patients failing to regain full function, with severe initial deficits predicting poor prognosis [1,2]. Long-term consequences include chronic instability, ankle contractures, and reliance on orthotics, necessitating secondary surgeries like nerve decompression in refractory cases [1]. 

Rationale for PPNR

During the correction of severe valgus deformity (>15-20°), the nerve, positioned in the concavity of the deformity, undergoes acute elongation, leading to microvascular compromise and neuropraxia. Stretch forces as low as 4-8% can disrupt intraneural blood flow, causing axonal injury [3,14]. Concurrent flexion contracture exacerbates this tension, increasing CPN palsy risk 12-fold compared to milder deformities [15].

Conventional lateral soft tissue releases, such as "pie-crusting", pose a risk of direct CPN injury due to the nerve's close proximity to surgical instruments [15]. PPNR mitigates this risk in several ways. It enables the direct visualization of the nerve during lateral releases, thereby preventing iatrogenic injury [7,10]. It also reduces stretch forces by allowing neurolysis, which increases nerve mobility and dissipates tension during deformity correction [5,7]. Additionally, it facilitates a more comprehensive lateral release without depth restrictions, optimizing soft tissue balancing and reducing the need for constrained implants [16].

PPNR eliminated CPN palsy in three studies (pooled analysis of 59 knees), despite severe valgus (mean: 18.6-31.3°) and high-risk comorbidities, including diabetes and prior lumbar or knee surgery [5,7,10]. Future development and validation of objective intraoperative nerve tension measurement techniques (e.g., strain gauges, ultrasonography) could further refine the indication for and adequacy of PPNR.

Surgical technique

PPNR is typically performed through a separate posterolateral incision before initiating TKA.

Incision and Exposure

A separate oblique or longitudinal incision is made approximately 3-6 cm in length, centered around the fibular neck, with a minimum skin bridge of 7-8 cm from the standard midline TKA incision to avoid wound complications. In Xu et al.'s approach, a 3 cm incision is made just lateral to the fibular head, identifying the CPN by first locating the biceps femoris and peroneus longus tendons [7]. Villa et al. emphasize marking all bony landmarks prior to exsanguination and using a supine position with lateral bump support to facilitate posterolateral access [10].

Neurolysis and Decompression

The CPN is identified along its course around the fibular neck and carefully freed from surrounding soft tissue structures. Dissection proceeds proximally and distally using electrocautery or scissors under loupe magnification to release all compressive fascial bands and intermuscular septa. Key structures include the fascia of the peroneus longus and the vertical intermuscular septum between the peroneus longus and extensor digitorum longus. Adequate decompression is confirmed when a finger can pass freely along the nerve both proximally and distally.

Intraoperative Adjustments

Xu et al. describe the intraoperative testing of nerve tension following deformity correction. If the nerve remains tight after initial release, further neurolysis is performed until full relaxation is achieved in extension [7]. The peroneus longus muscle belly may be partially detached to facilitate exposure and mobility if necessary.

TKA Procedure

After CPN decompression, TKA proceeds through a standard midline incision and medial parapatellar arthrotomy. Bone cuts are performed using standard alignment guides (intramedullary for the femur, extramedullary for the tibia), and lateral soft tissue balancing is achieved progressively. Lateral structures, including the iliotibial band, lateral collateral ligament, posterolateral capsule, and occasionally popliteus, are released using controlled pie-crusting techniques, often under the direct visualization of the decompressed nerve [17]. Xu et al. report that constrained implants were avoided due to improved balancing achieved through this technique [7].

Closure and Postoperative Protocol

Once final components are cemented and satisfactory gap balancing is confirmed, both incisions are closed in layers. Hemostasis is confirmed before closure, often with the use of tranexamic acid. Standard dressings are applied, and postoperative rehabilitation typically begins immediately with full weight-bearing and unrestricted knee motion [5,7,10].

Clinical outcomes

PPNR during TKA for valgus deformity has demonstrated consistently favorable results, particularly in high-risk patients. In individuals with severe valgus alignment, typically exceeding 15° and compounded by comorbidities such as diabetes, prior lumbar or knee surgeries, or concomitant flexion contractures, this technique has been associated with the complete preservation of postoperative motor and sensory nerve function [3].

Across multiple cohorts, postoperative neurologic outcomes were uniformly excellent. All patients maintained full muscle strength (grade 5) in the tibialis anterior, extensor hallucis longus, and peroneal muscles, along with intact cutaneous sensation over the lateral leg, dorsum of the foot, and first web space. No cases of transient or delayed-onset CPN palsy were reported postoperatively [3,11].

In addition to nerve protection, PPNR enables early functional rehabilitation. Patients were mobilized with full weight-bearing and an unconstrained range of motion on the first postoperative day, eliminating the need for knee flexion immobilization traditionally used to reduce nerve tension [18]. In a study by Makhdom et al., the average range of motion improved significantly, with flexion contractures reduced from 9° preoperatively to 1.2° postoperatively and knee flexion increasing from a mean of 103° to 111° (p<0.05) [5].

Importantly, PPNR enhanced intraoperative safety during lateral soft tissue balancing procedures such as pie-crusting of the iliotibial band, lateral collateral ligament, and posterolateral capsule. By placing the nerve under direct visualization, surgeons could proceed confidently with deeper releases without risking iatrogenic injury, a complication previously reported with standard techniques due to the CPN's proximity to surgical landmarks [3].

No adverse events have been directly attributed to the decompression procedure itself. The additional operative time was minimal (typically 15-30 minutes), and postoperative wound complications at the decompression site were rare and non-severe [18]. As such, PPNR appears to be a safe and effective adjunct in complex TKA for valgus knees, with growing support for its inclusion in surgical planning for appropriately selected high-risk patients. In a case series of four patients, Soundarrajan et al. reported significant postoperative improvements in both Knee Society Scores and functional scores, with no neurological deficits, supporting the role of PPNR in enabling safe correction and optimal functional recovery [19]. While pooled results across several studies demonstrate a 0% incidence of postoperative CPN palsy, it is crucial to acknowledge that this evidence stems primarily from small, non-randomized case series [3,5,7,10].

Complications

PPNR during TKA has been shown to be a safe adjunct procedure, with a very low rate of associated complications. Across published series involving high-risk valgus knees, no postoperative cases of CPN palsy have been reported following PPNR, suggesting strong protective efficacy in appropriately selected patients [3,11].

Minor complications directly attributable to the nerve release procedure are rare. The additional incision, typically 3-6 cm over the fibular neck with a maintained skin bridge from the main TKA incision, has not been associated with significant wound issues when standard precautions are followed [5,10]. Reports of hematoma or superficial wound problems have been isolated and generally mild, with no lasting sequelae or need for reoperation [18]. Although meticulous technique minimizes risk, potential long-term complications include the development of a sensory neuroma at the decompression site, arising from injury to small cutaneous branches during dissection; however, this remains a rare occurrence.

Importantly, the additional operative time required for PPNR is minimal, often ranging between 15 and 30 minutes, and does not interfere with the standard workflow of arthroplasty [5]. The use of loupe magnification, careful neurolysis, and release of fascial tunnels along the nerve's course are essential technical considerations to reduce iatrogenic trauma during dissection [5].

While current data are largely limited to small cohort studies and lack long-term follow-up, the absence of major complications, including infection, nerve injury, or wound breakdown, supports the safety profile of PPNR. Continued documentation of outcomes across larger and more diverse patient populations will be important to validate these findings and refine patient selection criteria.

Future directions

PPNR shows promise in mitigating nerve injury during TKA for severe valgus deformities, but critical knowledge gaps persist. Larger prospective studies are essential to validate efficacy across diverse populations and establish evidence-based angular deformity thresholds (e.g., valgus >15°±flexion contracture) for intervention [10]. Multicenter collaborations could overcome recruitment challenges for these relatively rare cases, enabling the robust analysis of long-term functional outcomes, cost-effectiveness, and implant survivorship [5]. Additionally, comparative trials contrasting PPNR versus alternative techniques (e.g., modified soft tissue releases or intraoperative neuromonitoring) are needed to define optimal nerve protection strategies [7]. Future work should also standardize surgical indications by integrating comorbidities (e.g., diabetes, prior neuropathy) and dynamic intraoperative assessments of nerve tension [7,10]. Finally, surgeon training protocols for CPN decompression, whether performed by arthroplasty or peripheral nerve specialists, require refinement to minimize technical complications and ensure reproducibility [5]. Addressing these priorities will solidify the role of PPNR in high-risk TKA.

## Conclusions

PPNR represents a promising adjunct in TKA for patients with severe valgus deformity and other high-risk factors for nerve injury. By addressing the anatomical and biomechanical vulnerabilities of the CPN during deformity correction, this approach facilitates safer soft tissue balancing, preserves nerve function, and enables earlier rehabilitation without increasing operative risk. Early clinical outcomes demonstrate consistent nerve protection and minimal complications, supporting its integration into surgical planning for select cases. As current evidence is based on small series, further high-quality, prospective research is necessary to refine patient selection criteria, validate long-term benefits, and define its role in modern arthroplasty practice.
